# The ectopic olfactory receptor OR7A17 regulates the proliferation and differentiation of human epidermal keratinocytes, and ginsenoside Rh3 acts as its antagonist

**DOI:** 10.1016/j.jgr.2025.100972

**Published:** 2026-01-05

**Authors:** Su Bin Han, Soyoon Baek, Eunbi Yu, Sae Woong Oh, Hyeyoun Kim, Seokhyeon Min, Gyeonghyeon Kim, Yeonsoo Kim, Koo Chul Kwon, Iseul Jo, Jae Youl Cho, In Ki Hong, Jongsung Lee

**Affiliations:** a Molecular Dermatology Laboratory, Department of Integrative Biotechnology, College of Biotechnology and Bioengineering, Sungkyunkwan University, Suwon-si, 16419, Gyunggi-do, South Korea; b Kolmar Korea, R&D Complex, 06800, Seoul, South Korea; c Molecular Immunology Laboratory, SEORIN COMPANY Co., Ltd., Chuncheon-si, Gangwon-do, South Korea; d Department of Cosmetics Engineering, Konkuk University, 05029, Seoul, South Korea; e Molecular Immunology Laboratory, Department of Integrative Biotechnology, College of Biotechnology and Bioengineering, Sungkyunkwan University, Suwon-si, 16419, Gyunggi-do, South Korea

**Keywords:** Cell proliferation, Differentiation, G-protein-coupled receptor, Mitogen-activated protein kinase, Olfactory receptor, Overexpression

## Abstract

**Background:**

Olfactory receptors perform diverse functions in many cell types and are ectopically expressed in human epidermis.

**Purpose:**

This study aimed to examine the role of OR7A17 in keratinocyte biology, its signaling pathway and the potential of ginsenosides as its antagonist.

**Methods:**

OR7A17 function was examined in stably OR7A17-overexpressing HaCaT cells using western blots, image analysis, flow cytometric and qPCR.

**Results:**

We found that OR7A17 overexpression promoted keratinocyte proliferation through MAPK signaling pathway, accompanied by elevated cAMP and cytosolic Ca^2+^ levels, which activated AP-1 and CRE. Moreover, blockade experiments showed that CNG, TRPV1, and TRPA1 channels mediated these Ca^2+^ signals. OR7A17 also promoted the PI3K/AKT pathway, activating NF-κB and driving G1/S cell cycle progression via GSK-3β inhibition and P70S6K activation. Conversely, OR7A17 overexpression repressed differentiation, as evidenced by reduced expression of early and late differentiation markers. Molecular docking and functional assays confirmed that ginsenoside Rh3 bound OR7A17 and antagonized its signaling, thereby inhibiting proliferation and restoring differentiation.

**Conclusion:**

In summary, OR7A17 promoted keratinocyte proliferation by activating PKA/MAPK, Ca^2+^/CREB, and PI3K-AKT-NF-κB pathways, while repressing differentiation. In addition, ginsenoside Rh3 functioned as an antagonist of OR7A17, counteracting these effects. These findings suggest OR7A17 as a therapeutic target for proliferation- and differentiation-related skin disorders such as atopic dermatitis, with ginsenoside Rh3 as a potential regulator for epidermal homeostasis.

## Graphical abstract

**Figure:**
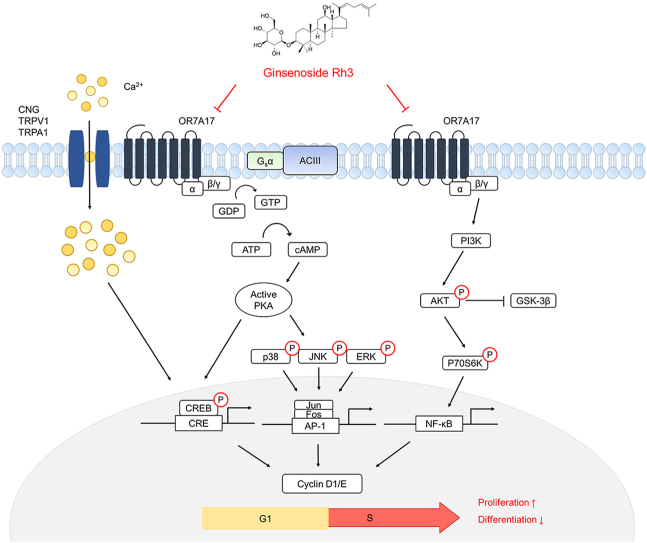

## 1 Introduction

Olfactory receptors (ORs) are members of the G-protein-coupled receptor (GPCR) family which were primarily smell receptors located in the nasal epithelium. However, several recent reports have indicated that ORs are not limited to the nasal epithelium but also play various physiological roles in several tissues, including the skin [[1], [2], [3]]. For example, as OR51E2 is highly upregulated in prostate carcinoma cells compared to normal healthy cells, these ORs have been considered as biomarkers for prostate tumors [4]. Furthermore, OR2AT4 activation in keratinocytes accelerates cell migration and proliferation, consequently promoting the wound-healing process [5]. These findings demonstrate that ectopic ORs can be essential diagnostic and therapeutic targets that have been underestimated thus far.

The skin acts as a barrier against environmental and physiological stresses. The epidermis, a stratified squamous epithelium, is continuously renewed by basal stem cells. Proliferation of transit-amplifying cells initiates keratinocyte differentiation, and as they migrate upward through the stratum spinosum and stratum granulosum, they ultimately transform into corneocytes that perform skin barrier functions. Fully differentiated corneocytes lose their cytoplasmic organelles and nuclei and are finally shed off via desquamation [6,7]. In the spinous layers, keratinocytes form desmosomal bundles of keratin filaments that provide structural integrity and protection from mechanical and non-mechanical stresses. The granular layers are primarily composed of involucrin, a major structural protein of the cell envelope (CE), which is upregulated at the onset of terminal differentiation and incorporated into CEs via cross-linking [8,9]. In the final stage, filaggrin expression predominates in the outermost layers, where it aggregates keratin proteins and promotes disulfide bond formation, leading to the establishment of a cornified CE [10].

Mitogen-activated protein kinases (MAPKs) are major cellular signaling pathways that convert extracellular signals into intracellular events [11,12]. They are activated by diverse stress-activating signals such as growth factors, interleukin-1, tumor necrosis factor-α, and UV radiation which trigger phosphorylation cascades involving p38, c-Jun N-terminal kinase (JNK), and extracellular signal-regulated kinase (ERK) [12,13]. These cascades transmit, amplify, and integrate signals from various stimuli, often through G protein-coupled receptors (GPCRs) mediated mechanisms [14]. One of the major downstream targets is activating protein-1 (AP-1), a transcription factor complex composed of Jun, Fos, and activating transcription factor subfamilies. AP-1 regulates several cellular processes including differentiation, apoptosis, and proliferation with its activity predominantly driven by MAPK-dependent induction of Jun and Fos transcription and by stimulation with 12-O-Tetradecanoylphorbol-13-acetate (TPA) [15].

GPCRs activate the phosphoinositide 3-kinase (PI3K)/protein kinase B (AKT) pathway, a crucial regulator of cellular growth, apoptotic process, and metabolism [16,17]. AKT, the serine/threonine protein kinase downstream of PI3K, regulates G protein-mediated nuclear factor kappa B (NF-κB) activation [18]. Through PI3K/AKT signaling, GPCRs promote NF-κB activity, thereby inducing cyclin D1 expression and facilitating G1/S transition, which drive cell cycle progression and proliferation [19,20].

Ectopic expression of ORs has been widely recognized, suggesting their potential as therapeutic targets for the development of several clinical drugs. However, the functions of ORs in non-olfactory tissues remain obscure. In the current study, we investigated the function of the OR7A17, an ectopically expressed OR in human keratinocytes, and its antagonist ligand by analyzing their impact on proliferation and differentiation using OR7A17-overexpressing HaCaT cell line and elucidating the underlying mechanisms.

## 2 Materials and methods

### 2.1 Materials

Antibodies against OR7A17 (PA5-71202) were obtained from Thermo Fisher Scientific. Antibodies against JNK, p-JNK, p-ERK 1/2, ERK 1/2, p-p38 MAPK, p38 MAPK, p-NF-κB, NF-κB, p-cyclic AMP-response element-binding protein (CREB), CREB, Cyclin D1, and Cyclin E were sourced from Santa Cruz Biotechnology. Additionally, antibodies against p-p70S6 kinase, p70S6 kinase, p-glycogen synthase kinase 3 beta (GSK-3β), GSK-3β, p-AKT, AKT, p21 Waf1/Cip1, and p-retinoblastoma (Rb) were acquired from Cell Signaling Technology. Antibodies for β-actin, anti-rabbit IgG, and anti-mouse IgG were purchased from Sigma-Aldrich.

### 2.2 Cell culture and compound treatment

The human keratinocyte cell line, HaCaT (ATCC), was cultured under previously described conditions [21]. OR7A17-HaCaT and mock-HaCaT cell lines were maintained under identical conditions media, with the addition of 10 μg/ml puromycin for cell selection. TPA (Sigma-Aldrich) was dissolved in DMSO and protected from light at −20 °C. Ginsenoside Rh3 (ChemFaces) was dissolved in DMSO and were treated for 48 h, with medium refreshed every 24 h.

### 2.3 Western blotting analysis

Western blotting analysis were conducted as described previously [22]. Briefly, cells were harvested, lysed in RIPA buffer containing protease inhibitors (Cell Signaling Technology), and clarified by centrifugation. Protein concentrations were measured using the BCA assay (Thermo Fisher Scientific). Equal amounts of proteins were separated by SDS-PAGE, transferred onto PVDF membranes (Bio-Rad), and probed with the indicated primary and HRP-conjugated secondary antibodies. Blots were visualized using an ECL detection system (Bio-Rad).

### 2.4 Analysis of mRNA levels using quantitative PCR (RT-qPCR)

RNA sample was prepared following the previously described conditions [21]. Total RNA was extracted and reverse-transcribed into cDNA using TOPscript™ RT DryMix (Enzynomics, Korea). Gene expression was quantified by qPCR and normalized to GAPDH as an internal control. PCR products were analyzed by agarose gel electrophoresis. The primer sequences that were used in this study are listed in Supplementary Table 1.

### 2.5 Luciferase reporter and β-Galactosidase activity assays

Luciferase and β-Galactosidase activity assay were conducted as previously described [23]. NF-κB, CRE, and AP-1 promoter-luciferase plasmids (Stratagene) were transfected into cells using polyethyleneimine (Polyscience). Reporter activity was measured with a luciferase assay system (Promega) and normalized to β-galactosidase activity.

### 2.6 5-Ethynyl-2′-deoxyuridine (EdU) incorporation assay

Cell proliferation was assessed using the Click-iT™ EdU imaging kit (Invitrogen) according to the manufacturer's instructions [21]. Labeled cells were fixed, permeabilized, stained with the reaction cocktail. The nucleus was stained with Hoechst 33342 and fluorescent images were visualized using a confocal microscope (Zeiss LSM 700).

### 2.7 Cell proliferation analysis

Cell proliferation analysis was applied using a BrdU ELISA kit (Invitrogen) and CellTiter Glo® 2.0 reagent (Promega) according to the manufacturer's instruction [24]. Cells (5 × 10^3^) were cultured in 96-well plates and treated under experimental conditions when reaching 60 % confluence.

### 2.8 Fluo-4 Ca^2+^ influx assay

Ca^2+^ influx assay was conducted according to described conditions [22]. We used the Fluo-4 NW Ca^2+^ assay kit (Invitrogen) and used 96-well with black wall/clear bottom plates. Culture medium was discarded, and the cells were stained using Fluo-4 NW Ca^2+^ reagent. The fluorescence was measured using a Synergy HTX (BioTek) at an excitation wavelength of 494 nm and an emission wavelength of 516 nm.

### 2.9 Flow cytometry analysis

Flow cytometry was performed by pretreating cells with TPA. The cells were washed with PBS and then fixed with chilled 70 % ethanol. The cells were rinsed with chilled PBS and centrifuged at 1000×*g* for 5 min and suspended in PBS at a concentration of 50 μg/mL propidium iodide and 100 μg/mL RNase A and incubated in the dark. Finally, the distribution of cell cycle stages and DNA content was assessed using a CytoFLEX (Beckman Coulter).

### 2.10 Clonogenic assay

In 6-well plates 5 × 10^2^ cells per well were plated and maintained, with the culture medium being refreshed on a two-day interval. The cells were incubated at 37 °C for a period of 10–14 days to promote colony formation. The cells were rinsed with PBS, fixed with methanol, and stained with a 0.5 % crystal violet solution (Sigma-Aldrich) in the dark. The crystal violet solution was removed. The cells were then gently washed with running tap water. The plates were left to air-dry at and the number of formed-colonies was counted.

### 2.11 In silico analysis

Molecular docking studies were performed to predict the binding affinity between OR7A17 and selected ligand candidates using the AutoDock software [25,26]. The 3D structure of OR7A17 was obtained from an Alpha-Fold Protein Structure Database. Ligand structures were downloaded from the PubChem database. The compound identifiers of ligand candidates were listed in Supplementary Table 2. Prior to docking, all ligands were energy-minimized, and polar hydrogens and Kollman charges were added to the protein structure. The docking grid was on the predicted ligand-binding pocket of OR7A17. The resulting docking poses were ranked based on binding energy scores; the lowest-energy conformation was considered the highest binding affinity.

### 2.12 Lentiviral transduction

Lentiviral transduction for OR7A17 knockdown was carried out according to a previously established protocol [22]. The lentiviral backbone vector pLKO.1 (Addgene plasmid # 8453) puro was kindly provided by Bob Weinberg [27]. The packing plasmid psPAX2 (Addgene plasmid # 12260) and the envelope plasmid pMD2.G (Addgene plasmid # 12259) were gifts from Didier Trono. For silencing of OR7A17, a predesigned shRNA sequence (CCCTGAATTCCTTGTCACAAA) was obtained from Sigma-Aldrich. Viral particles (3.5 μL/mL), resuspended in 200 μL PBS, were added to HaCaT cells together with polybrene (10 μg/mL). Following 24 h of incubation, the culture medium was exchanged for fresh medium supplemented with puromycin (10 μg/mL), and subsequent assays were performed 24 h later.

### 2.13 CETSA (cellular thermal shift assay)

CETSA was performed according to a previously established protocol with minor modifications [28]. For melting curve CETSA, cells were treated compounds for 1h and incubated at 37 °C prior to heat challenge. Then the cell suspensions were aliquoted into PCR tubes and exposed to a temperature gradient, followed by rapid cooling and repeated freeze and thaw cycles to lyse cells. Soluble protein fractions were obtained after centrifugation and analyzed by immunoblotting to quantify remaining non-aggregated target protein. For isothermal dose-response CETSA, cells were incubated with serially diluted compounds and heated at a predetermined temperature, after which soluble protein levels were assessed by immunoblotting.

### 2.14 Statistical analysis

The data were validated by performing at least three independent experiments. They are presented as mean ± standard deviation. Statistical analysis was conducted using Student's *t*-test between two groups and one-way ANOVA when multiple groups were compared. Differences in means were considered statistically significant if the p-value was less than 0.05.

## 3 Results

### 3.1 Effect of OR7A17 on HaCaT cells proliferation

**Fig. 1:**
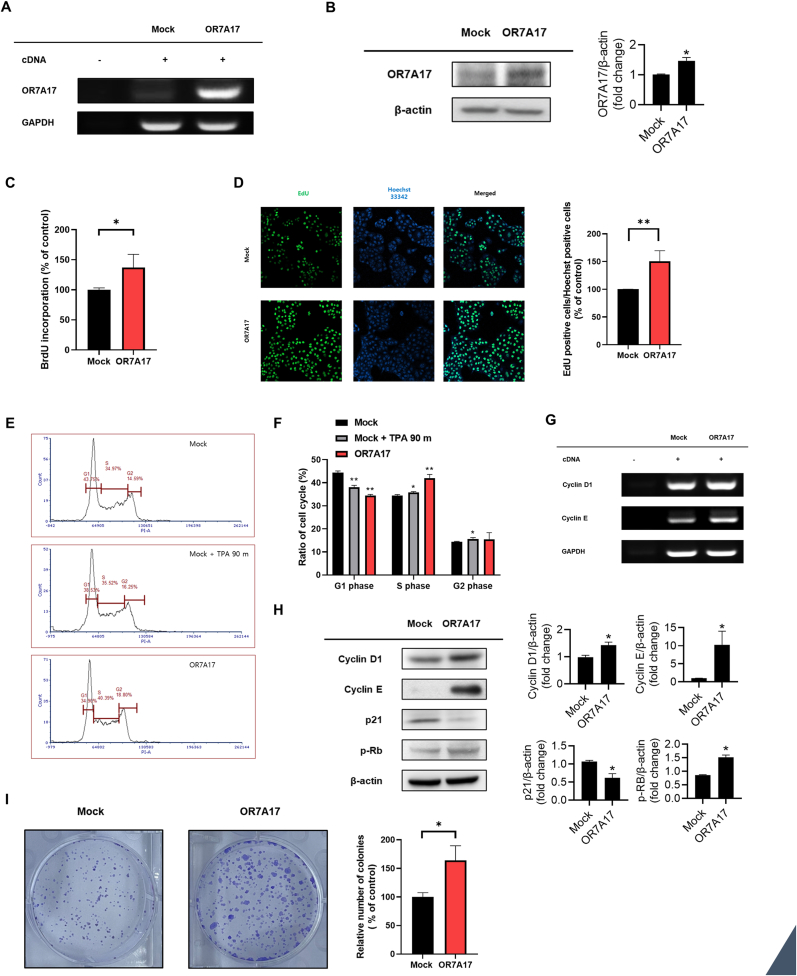
OR7A17 overexpression increases proliferation of HaCaT cells. (A) OR7A17 expression levels in HaCaT cells were measured by RT-qPCR and (B) Western blot analysis. (C) The effects of OR7A17 on proliferation were determined by BrdU ELISA and (D) EdU incorporation assays. (E) Flow cytometry analysis by PI staining. Mock-HaCaT cells were incubated with 10 nM TPA for 90 min. Representative histogram of the gated cells in the G1, S, and G2 phases. (F) The graph illustrates the percentage of cells in each phase (G) RT-qPCR was performed to determine mRNA levels of cyclins D1 and E. (H) Western blotting was performed to determine the expression levels of cyclin D1, cyclin E, p21, and p-Rb. (I) A clonogenic assay was performed on OR7A17-HaCaT cells. The images were taken following a two-week incubation of the cells (∗p < 0.05 vs. Mock, ∗∗p < 0.01 vs. Mock).

As previous studies have reported that ectopically expressed ORs enhance cell proliferation in keratinocytes [3,5], we examined the proliferative effect of OR7A17 overexpression in HaCaT cells. Prior to these assays, we performed both RT-PCR (Fig. 1A) and western analyses (Fig. 1B) to confirm OR7A17 expression in OR7A17-overexpressing HaCaT cell line established through the transduction of the LvCMV-OR7A17 vector into HaCaT cells [21]. Using a BrdU ELISA assay, we confirmed the proliferative effect of OR7A17 (Fig. 1C). Similarly, an EdU incorporation assay was performed to confirm OR7A17-mediated proliferation. Compared to the mock-transduced cells, OR7A17-overexpressing cells exhibited 50.6 % higher proliferative rate (Fig. 1D). Furthermore, we examined the cell cycle progression mediated by OR7A17 in human keratinocytes using flow cytometry. As shown in Fig. 1E and F, OR7A17-overexpressing cells showed a lower proportion of cells in the G1 phase and a higher proportion of cells in the S phase, indicating an active G1-to-S phase transition and DNA synthesis. To further elucidate the specific cell-cycle regulatory genes that promote the G1/S transition, the expression levels of cyclins D and E were evaluated by RT-PCR. We found that their expressions were significantly increased in OR7A17-overexpressing cells (Fig. 1G). Consistent with the mRNA levels, OR7A17-overexpressing cells showed increased protein expression of cyclins D and E. While the protein levels of p-Rb increased, that of p21 decreased in OR7A17-overexpressing cells (Fig. 1H). Furthermore, we analyzed the proliferative effect of OR7A17 overexpression using a clonogenic assay (Fig. 1I). OR7A17-overexpressing cells showed a significant increase in colony formation compared with the mock group. Collectively, these results indicate that OR7A17 overexpression strongly enhances the proliferative potential of human epidermal keratinocytes.

### 3.2 OR7A17 overexpression activates both MAPK and CREB signaling pathways

**Fig. 2:**
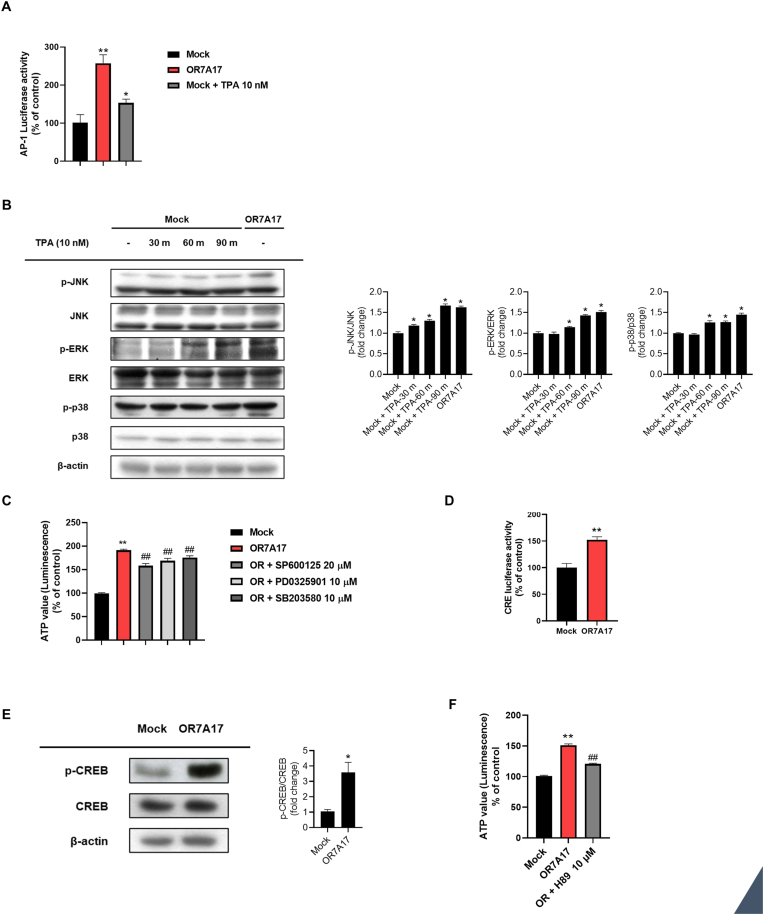
OR7A17 overexpression elicits MAPK and CREB signaling activation. (A) Cells were transfected with an AP-1 promoter luciferase reporters and incubated for 4 h. Mock-HaCaT cells were then incubated with 10 nM of TPA for 24 h and included in a luciferase reporter assay (B) Mock-HaCaT cells were treated with 10 nM TPA for the indicated time periods (30, 60, or 90 m). After the treatment period, the cells were collected with OR7A17-HaCaT cells and analyzed for the phosphorylation of JNK, ERK, and p38 by Western blot analysis. (C) Cell proliferation was evaluated by CellTiter-Glo 2.0 assay. (D) Cells were transfected with CRE promoter luciferase reporters and incubated for 4 h and subjected to a luciferase reporter assay. (E) CREB phosphorylation was analyzed by Western blot analysis. β-actin was used as the control (F) Cell titer assay after treating with H89 for 24 h (∗p < 0.05 vs. Mock, ∗∗p < 0.01 vs. Mock, ##p < 0.01 vs OR7A17).

MAPK signaling pathways play pivotal roles in cellular signal transduction by orchestrating various cellular responses, including cell survival and proliferation [[11], [12], [13]]. AP-1 activation regulates cellular proliferation and is induced by MAPK signal transduction [29]. To further examine the involvement of OR7A17 in the MAPK signaling, we performed an AP-1-luciferase reporter assay in OR7A17-overexpressing HaCaT cells. The results showed that OR7A17-overexpressing cells showed enhanced AP-1 luciferase reporter activity, indicating that OR7A17 is involved in MAPK activation (Fig. 2A). Additionally, in the Western blot analysis for MAPKs, we found a significant increase in the phosphorylation of p38, ERK, and JNK in OR7A17-overexpressing HaCaT cells (Fig. 2B). Treatment with TPA for a short period was used to induce the phosphorylation of three MAPK factors as positive controls. Furthermore, treatment of OR7A17-overexpressing cells with inhibitors (SP600125 [a JNK inhibitor], PD0325901 [an ERK inhibitor], and SB203580 [a p38 inhibitor]) showed a significant decrease in the cell titer assay, indicating that MAPKs are involved in OR7A17-induced proliferation (Fig. 2C).

GPCR stimulation enhances the activity of the cAMP/protein kinase A (PKA) pathway, thereby augmenting the phosphorylation of CREB [30]. We examined CRE activation using a CRE-luciferase reporter assay. The CRE luciferase reporter activity was enhanced in OR7A17-expressing HaCaT cells (Fig. 2D). Moreover, CREB phosphorylation was elevated in HaCaT-OR7A17 cells (Fig. 2E). Furthermore, using the cell titer assay, we found a substantial decrease in ATP levels following 24 h treatment with H89, a PKA inhibitor (Fig. 2F). These findings indicate that OR7A17 promotes proliferation by activating the MAPK and CREB signaling pathways.

### 3.3 OR7A17 induces proliferation through Ca^2+^ signaling via CNG, TRPV1, and TRPA1 channels

**Fig. 3:**
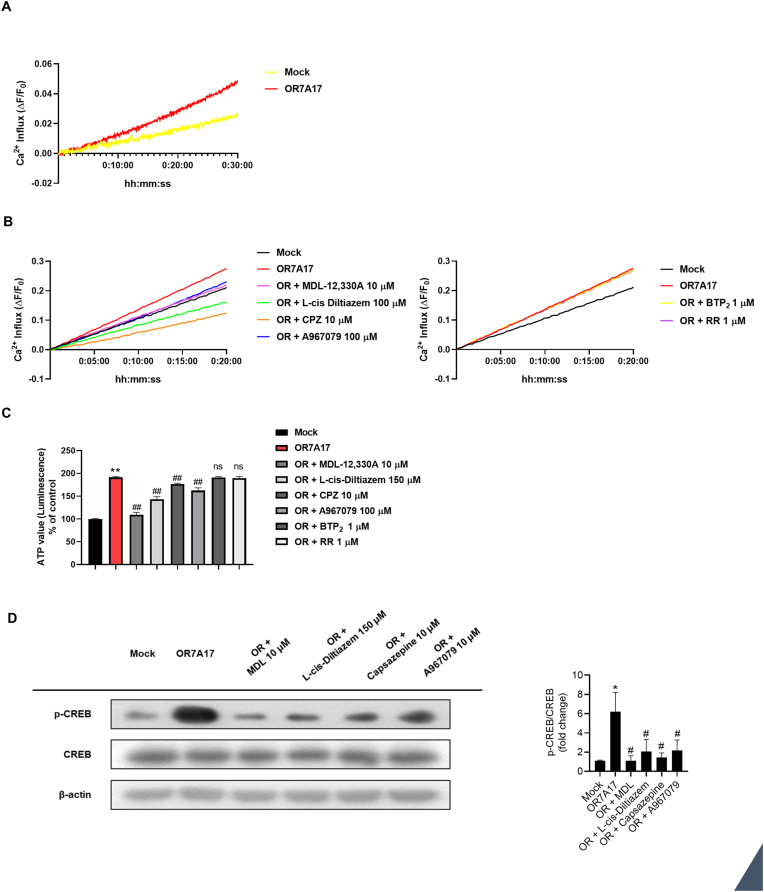
Activation of the Ca^2+^ signaling pathway induces cell proliferation. (A, B) Changes in Ca^2+^ influx after indicated treatments were observed using Fluo-4 probe. (C) Cell viability was evaluated by CellTiter-Glo 2.0 assay. Cells were incubated in the presence of MDL-12,330A (10 μM), L-cis Diltiazem (150 μM), capsazepine (10 μM), A967079 (100 μM), BTP_2_ (1 μM), and ruthenium red (1 μM) for 24 h. (D) Expression of p-CREB were analyzed by western blotting. β-actin was used as the control for whole-cell lysates (∗∗p < 0.01 vs. Mock, ##p < 0.01 vs OR7A17).

Studies have shown that GPCR activation induces a cascade of signaling systems that regulate cytosolic calcium dynamics [31]. Therefore, we conducted a calcium influx assay to assess whether OR7A17 overexpression is involved in the modulation of Ca^2+^ entry. As shown in Fig. 3A, OR7A17-overexpressing HaCaT cells showed greater calcium influx than the mock control. To evaluate the effect of OR7A17 on calcium signaling, we conducted a series of experiments using various calcium inhibitors. As shown in Fig. 3B, the Ca^2+^ influx assay revealed a notable reduction in Ca^2+^ influx upon treatment with MDL-12,330A, an adenylyl cyclase inhibitor responsible for cAMP generation. Additionally, inhibitors targeting specific channels, such as L-cis diltiazem (CNG channel), capsazepine (TRPV1), and A967079 (TRPA1), led to a notable reduction in Ca^2+^ influx in OR7A17-overexpressing HaCaT cells. In contrast, the ORAI1 antagonist, BTP_2_, and the TRPV3 antagonist, ruthenium red, showed no significant alterations, suggesting selective modulation of calcium influx in OR7A17-overexpressing HaCaT cells. Specifically, while the CNG, TRPV1, and TRPA1 channels regulate OR7A17-mediated signaling, ORAI1 and TRPV3 are not involved in OR7A17 signaling. To confirm the findings from the calcium influx assay (Fig. 3B), a cell titer assay was conducted for 24 h using the same inhibitors (Fig. 3C). Consistent with the calcium assay results (Fig. 3B), MDL-12,330A and channel-specific inhibitors, including L-cis diltiazem, capsazepine, and A967079, induced a substantial decrease in cell viability. Conversely, BTP_2_ and ruthenium red did not exhibit any discernible impact on cell viability.

Calcium signaling is known to activate pathways leading to CREB phosphorylation, a critical event in the control of gene expression related to cellular proliferation [32,33]. Therefore, we investigated the status of CREB phosphorylation by western analysis. As anticipated, CREB phosphorylation consistently diminished following treatment with the inhibitors (MDL-12,330A, L-cis diltiazem, capsazepine, and A967079) (Fig. 3D). These observations indicate that Ca^2+^ mobilization operates upstream of CREB phosphorylation in OR7A17-mediate signaling and suggest that OR7A17-Ca^2+^-CREB regulates cell proliferation.

### 3.4 The PI3K/AKT/NF-κB signaling pathway regulates OR7A17-induced proliferation

**Fig. 4:**
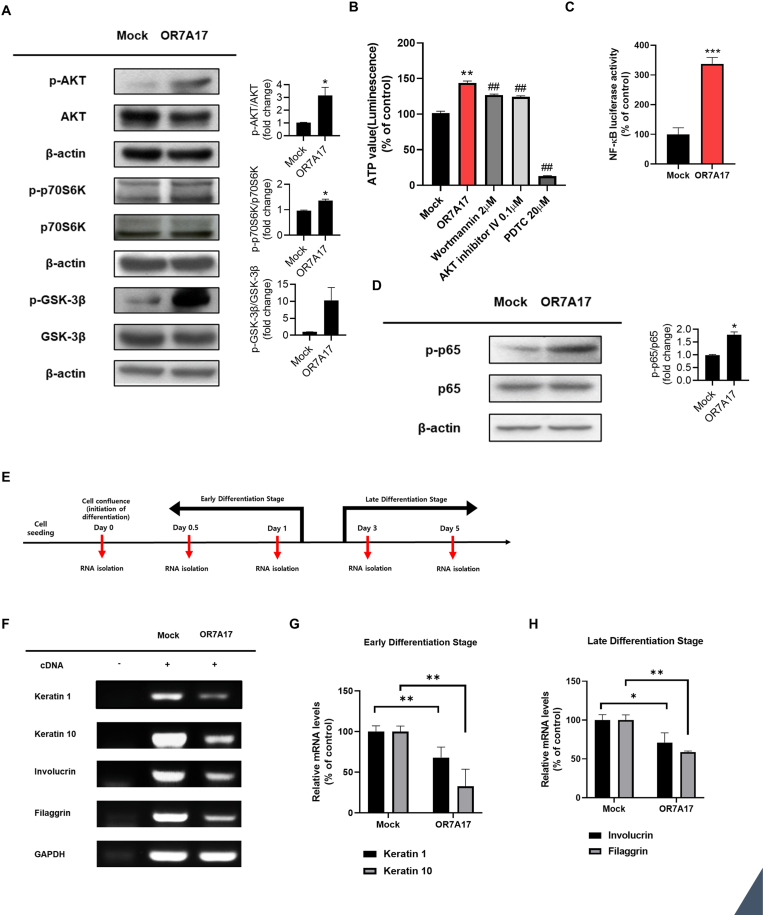
OR7A17 induces cell proliferation through PI3K/AKT/NF-κB signaling pathway and reduced differentiation. (A) Phosphorylation levels of AKT, P70S6K, and GSK-3β were measured by western blotting. β-actin was used as a loading control. (B) Cell viability was evaluated through CellTiter-Glo 2.0 assay. Cells were incubated in the presence of wortmannin (2 μM), AKT inhibitor IV (0.1 μM), and pyrrolidine dithiocarbamate (PDTC; 20 μM) for 24 h. (C) Luciferase reporter assay after transfection with NF-κB luciferase plasmid. (D) Expression of p-p65 was analyzed by western blotting. (E) OR7A17-HaCaT and Mock-transduced HaCaT cells were cultured until they reached the confluent state (Day 0). Subsequently, a 5-day differentiation period was implemented, and RNA was isolated at Day 0, Day 0.5, Day 1, Day 3, and Day 5 for RT-qPCR analysis. (F) mRNA levels of keratin 1, keratin 10, involucrin, and filaggrin were analyzed by RT-qPCR. GAPDH was used as the loading control. (G, H) Densitometric analysis was performed for three different experiments (∗p < 0.05 vs. Mock, ∗∗p < 0.01 vs. Mock, ##p < 0.01 vs OR7A17).

GPCRs directly activate the PI3K/AKT pathway, which plays a crucial role in regulating cell proliferation and survival [16]. To examine the involvement of the PI3K/AKT/NF-κB pathway in OR7A17-induced cell proliferation, Western blot analysis was performed. Phosphorylation levels of AKT, p70 ribosomal protein S6 kinase (P70S6K), and GSK-3β were increased in OR7A17-overexpressing HaCaT cells (Fig. 4A). Next, we determined whether OR7A17-induced proliferation is dependent on PI3K/AKT/NF-κB signaling by conducting cell titer assay. As shown in Fig. 4B, treatment with a.PI3K inhibitor (wortmannin), AKT inhibitor IV, and NF-κB inhibitor (PDTC) on OR7A17-overexpressing cells for 24 h significantly reduced the cell proliferation. To further examine the involvement of NF-κB signaling, NF-κB luciferase reporter assay was conducted. The luciferase activity of NF-κB was expectedly increased in OR7A17-overexpressing cells (Fig. 4C). Additionally, western analysis confirmed increased phosphorylation levels of p65 (Fig. 4D). Collectively, these suggest that OR7A17 overexpression promotes HaCaT cell proliferation through the PI3K/AKT/NF-κB signaling pathway.

### 3.5 Analysis of keratinocytes differentiation markers in OR7A17-overexpressing HaCaT cells

To establish a robust epidermal structure, keratinocytes undergo continuous cell division, which leads to their differentiation into a stratified epithelium [34]. Therefore, we analyzed the mRNA expression of differentiation barrier markers in epithelial layers. As indicated in Fig. 4F and G, the mRNA levels of keratin 1 and keratin 10 were significantly reduced in OR7A17-overexpressing HaCaT cells from days 0–1, which are considered early differentiation states. Furthermore, involucrin and filaggrin mRNA levels decreased in more progressed differentiation states from days 3–5 (Fig. 4F–H). Collectively, these suggest that OR7A17 overexpression in HaCaT cells suppresses epidermal differentiation.

### 3.6 Ginsenoside Rh3 acts as a possible antagonist for OR7A17

**Fig. 5:**
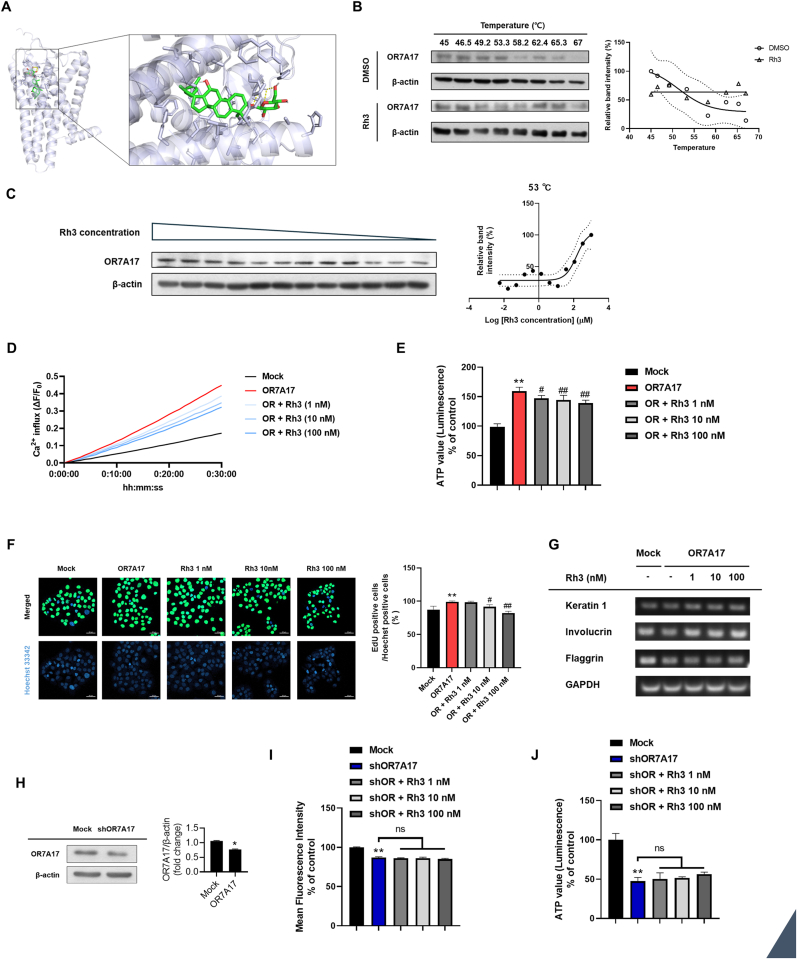
Ginsenoside Rh3 acts as a possible antagonist for OR7A17. (A) Docking analysis of ginsenoside Rh3 with the OR7A17 binding pocket. The interaction is illustrated using a 3D cartoon model (predicted binding affinity: 9.9 kcal/mol) (B) CETSA melting curves showing thermal stabilization of7A17 in Rh3-treated OR7A17-overexpressing cells compared with DMSO-treated OR7A17-overexpressing cells. The final concentration of Rh3 was 100 μM. (C) Isothermal dose-response CETSA demonstrated a dose-dependent stabilization of OR7A17 by Rh3. (D) Intracellular calcium level was measured using Fluo-4 probe. (E) Cell viability was evaluated through CellTiter-Glo 2.0 assay. (F) The effects of ginsenoside Rh3 on proliferation were determined by EdU incorporation assays. (G) mRNA levels of keratin 1, keratin 10, involucrin, and filaggrin were analyzed by RT-qPCR. (H) The protein level of OR7A17 was measured by Western blot analysis. β-actin was used for loading control. (I) Intracellular calcium level was measured using Fluo-4 probe. MFI (mean fluorescence intensity) was presented. (J) Relative cell viability levels of Mock, OR7A17 knockdown cell line, and Rh3 treated OR7A17 knockdown cell line were evaluated through CellTiter-Glo 2.0 assay (∗p < 0.05 vs. Mock, ∗∗p < 0.01 vs. Mock, #p < 0.05 vs OR7A17, ##p < 0.01 vs OR7A17).

Ginsenosides have been known to exert various physiological activities in the skin [[35], [36], [37]]. Therefore, we examined the interaction between OR7A17 and ginsenosides using the AutoDock virtual ligand screening software [25,26]. First, screening using the AutoDock method was conducted using the ambrein, which is known as the ligand molecule of OR7A17 [38]. Based on the result obtained with AutoDock, the highest binding affinity of ambrein was −10.0 kcal/mol. After verifying the receptor-ligand affinity using ambrein, the binding affinities of ginsenosides and OR7A17 were measured (Table 1). Among the ginsenosides, the highest binding affinity of OR7A17 and ginsenoside Rh3 using AutoDock Tools was −9.9 kcal/mol (Fig. 5A). To further validate that ginsenoside Rh3 directly interacts with OR7A17, we performed CETSA. Exposure to Rh3 led to enhancement in the thermal stability of OR7A17 (Fig. 5B). In OR7A17-overexpressing cells, Rh3 maintained a higher level of soluble OF7A17 following heat treatment, whereas cells treated with vehicle control showed no notable stabilization. Consistently, the isothermal dose-dependent CETSA revealed a concentration-dependent increase in soluble OR7A17 upon Rh3 treatment (Fig. 5C), supporting the notion that Rh3 interacts with ginsenoside Rh3. Subsequently, we conducted a calcium influx assay to assess whether ginsenoside Rh3 is involved in the modulation of Ca^2+^ entry. As shown in Fig. 5D, in OR7A17-overexpressing cells, treatment with ginsenoside Rh3 significantly decreased intracellular Ca^2+^ influx compared with untreated OR7A17-overexpressing cells, reversing the increases observed relative to mock-transduced cells. Furthermore, treatment of OR7A17-overexpressing cells with ginsenoside Rh3 showed a concentration-dependent decrease in the cell titer assay, indicating that it counteracted OR7A17-induced proliferation (Fig. 5E). Consistent with the cell titer assay, ginsenoside Rh3 reversed the enhanced proliferative rate observed in OR7A17-overexpressing cells, restoring proliferation to levels comparable with mock controls (Fig. 5F). In addition to proliferative changes, we examined the influence of ginsenoside Rh3 on differentiation. Prior experiments demonstrated that OR7A17 overexpression markedly reduced the expression of differentiation barrier markers (Fig. 4F–H). Furthermore, ginsenoside Rh3 treatment restored the expression of these markers, indicating that ginsenoside Rh3 opposed the OR7A17-mediated suppression of differentiation (Fig. 5G).

**Table 1:** Virtual docking screening between OR7A17 and Ginsenosides *in silico*.

Compounds	Affinity (kcal/mol)	Ligand Efficiency
Ambrein	−10.0	−0.32
Ginsenoside Rh3	−9.9	−0.23
Ginsenoside Rb2, Rb3, Rc	−9.6	−0.13
Ginsenoside Rb1	−9.5	−0.12
Ginsenoside CK	−9.2	−0.21
Ginsenoside Rg2	−8.9	−0.16
Ginsenoside Rh2	−8.8	−0.20
Ginsenoside Re	−8.6	−0.13
Ginsenoside F2	−8.5	−0.15
Ginsenoside Rd	−8.3	−0.13
(−)-Ambroxide	−8.1	−0.43
Ginsenoside Rg3	−8.1	−0.15
Ginsenoside Rg1	−7.7	−0.14

To determine whether the activity of ginsenoside Rh3 was mediated through OR7A17, we established OR7A17-knockdown HaCaT cell line (Fig. 5H). Compared with mock control, OR7A17-knockdown cells exhibited a reduction in intracellular Ca^2+^ mean fluorescence intensity (MFI) and decreased cell viability (Fig. 5I and J). Notably, the inhibitory effects of ginsenoside Rh3 were abrogated in OR7A17-knockdown cells (Fig. 5G and H), supporting that ginsenoside Rh3 acts as an antagonist of OR7A17.

## 4 Discussion

Ectopic expression of ORs and their distinctive functions have been widely reported. Beyond odor detection, ORs regulate cancer cell proliferation, migration, and survival with receptors such as OR2B6, OR51E2, OR51B4, OR10H1, and OR1A1 associated with proliferative processes [[39], [40], [41], [42], [43]]. In the skin, OR2AT4, OR2A4/7, OR41E2, and OR10G7 have been linked to cellular proliferation [3,5,44,45].

Ors, as GPCRs, are expressed primarily in olfactory sensory neurons [46], however, their expression has also been documented in various tissues, including the skin, lungs, and brain, where they perform physiological roles apart from olfaction [1,2,4,5,45]. Despite these findings, comprehensive analyses of orphan ORs in the skin remain unexplored. We have previously demonstrated the physiological functions of the novel OR, OR7A17, which have not been previously investigated in human keratinocytes [21]. In this study, we investigated the exact mechanism of action of OR7A17 by exploring its role in cellular proliferation and differentiation to modulate epidermal homeostasis.

Previous studies have shown that several ORs ectopically expressed in HaCaT cells are involved in keratinocyte proliferation [1,3,5]. Cell cycle transition from G1 to S phase is controlled by cyclin D1 and cyclin E, which form complexes with cyclin dependent kinase (CDK) 4 and CDK2 [47]. The CDK4/cyclin D1 complex drives cells into the G1 phase and phosphorylates Rb, whereas the CDK2-cyclin E complex inactivates Rb. Phosphorylated Rb releases E2F transcription factors, initiating the G1/S transition [48,49]. Our study demonstrated that OR7A17 overexpression promoted keratinocyte proliferation, as shown by BrdU and EdU assays, increased S/G2 phase populations, and elevated levels of cyclin D1, cyclin E, and phosphorylated Rb. These indicate that OR7A17 regulates the proliferation of human epidermal keratinocytes.

MAPKs, critical downstream molecules of GPCRs, regulate proliferation, differentiation, and skin barrier function [12]. OR7A17-overexpressing cells exhibited AP-1 activation along with phosphorylation of p38, ERK, and JNK. These results suggest that OR7A17 enhances keratinocyte proliferation through MAPK signaling pathway.

GPCRs activation triggers dissociation of G proteins, leading to adenylyl cyclases (ACs) activation, cAMP production, and subsequent PKA-mediated phosphorylation of CREB, which regulates cell growth, differentiation, and metabolism [30,32]. In OR7A17-overexpressing cells, CREB was significantly activated. GPCR also regulate cytosolic Ca^2+^ dynamics, which are essential for proliferation and differentiation [31,32]. Blockage experiment revealed that while OR7A17 activated Ca^2+^ signaling through CNG, TRPV1, and TRPA1, the ORAI1 and TRPV3 channels were not activated. We also found that inhibition of these channels reduced CREB phosphorylation, linking OR7A17-mediated Ca^2+^ influx through CNG, TRPV1, and TRPA1 to CREB activation and proliferation.

In addition, GPCRs are known to activate the PI3K/AKT pathway through Gα and Gβγ subunits, thereby regulating cell survival, metabolism, and proliferation [16,17]. Activation of PI3K initiates phosphorylation of AKT and its downstream kinases, including GSK-3 and p70S6K, and have also been implicated in NF-κB control [17,50]. In our study, OR7A17 overexpression markedly increased phosphorylation of AKT, GSK-3β, p70S6K, and NF-κB. Additionally, treatment with inhibitors targeting PI3K, AKT, or NF-κB reduced cell viability, indicating that OR7A17 promotes keratinocyte proliferation through PI3K/AKT/NF-κB signaling.

Epidermal homeostasis relies on the balance between keratinocyte proliferation and differentiation [10,51]. During terminal differentiation, keratinocytes assemble structural proteins to form a cornified CE [9]. In this study, OR7A17-overexpressing HaCaT cells showed reduced expression of early (Keratin 1, Keratin 10) and late (Involucrin and Filaggrin) differentiation markers, indicating OR7A17 inhibits epidermal differentiation while enhancing proliferation.

Atopic dermatitis (AD), a chronic inflammatory skin disorder, is characterized by abnormal keratinocyte differentiation and impaired epidermal barrier function. Our findings support that OR7A17 was associated with enhanced cellular proliferation and suppression of keratinocyte differentiation, both of which are hallmarks observed in the lesional skin of AD patients. Consistent with our expectation, OR7A17 expression is significantly elevated in AD patients [45]. Importantly, we demonstrated that ginsenoside Rh3, a bioactive compound derived from ginseng, functions as an antagonist of OR7A17, suppressing OR7A17-derived hyperproliferation and restoring differentiation in keratinocytes. These results highlight the therapeutic potential of ginsenoside Rh3 in AD.

In conclusion, these findings indicate that OR7A17 promotes epidermal keratinocyte proliferation via PKA/MAPK, Ca^2+^/CREB, and PI3K-AKT-NF-κB pathways, thereby suppressing differentiation. Furthermore, we identified ginsenoside Rh3 as a functional antagonist of OR7A17, suggesting that OR7A17 could serve as a potential therapeutic target for proliferation- and differentiation-related diseases such as AD and ginsenoside Rh3 could be also used as an agent for epidermal homeostasis.

## CRediT authorship contribution statement

S.B.H., S.B., E.Y., H.K., S.M., G.K., Y.K., K.C.K., I.J., J.Y.C., I.K.H., and J.L. conceptualization, methodology, formal analysis, writing of the original draft; H.K., S.M., G.K., K.C.K., I.J. conceptualization, methodology; J.Y.C., I.H., and J.L. funding acquisition, conceptualization, methodology, supervision, writing review, and editing. All the authors have read and agreed to the published version of the manuscript.

## Data availability statement

Data used to support the findings of this study are available from the corresponding author upon request. The data presented in this study are available in this article.

## Funding

This research was supported by a grant from the Basic Science Research Program through the 10.13039/501100003725National Research Foundation of Korea (NRF) funded by the Ministry of Science and Technology Information and Communication (grant no. RS-2023-00246887) and a grant from Kolmar Korea.

## Declaration of competing interest

The authors declare no conflicts of interest. The funders had no role in the study design; collection, analyses, or interpretation of data; writing of the manuscript; or decision to publish the results.
