# Supplementary material for: The ectopic olfactory receptor OR7A17 regulates the proliferation and differentiation of human epidermal keratinocytes, and ginsenoside Rh3 acts as its antagonist
Source: J Ginseng Res. 2026 Jan 5;50(2):100972. doi: 10.1016/j.jgr.2025.100972 (PMC12959293; doi:10.1016/j.jgr.2025.100972)
Supplement: Multimedia component 1 [file mmc1.docx]

Supplementary Table 1. List of primers synthesized for RT-qPCR.

| **Gene Name** |  | **Sequence (5’ - 3’)** |
| --- | --- | --- |
| OR7A17 | Forward | TGG AAC CAG AGA ATG ACA CAG G |
|  | Reverse | GAT GAG CAG ATT CCC GAG CA |
| Cyclin D1 | Forward | GCT GCG AAG TGG AAA CCA TC |
|  | Reverse | CCT CCT TCT GCA CAC ATT TGA A |
| Cyclin E | Forward | GCC AGC CTT GGG ACA ATA ATG |
|  | Reverse | CTT GCA CGT TGA GTT TGG GT |
| Keratin 1 | Forward | AGA GTG GAC CAA CTG AAG AGT |
|  | Reverse | ATT CTC TGC ATT TGT CCG CTT |
| Keratin 10 | Forward | ATG TCT GTT CGA TAC AGC TCA AG |
|  | Reverse | CTC CAC CAA GGG AGC CTT TG |
| Involucrin | Forward | GCA GGA CGG ACA ACT AAA AC |
|  | Reverse | GCT GGT TGA ATG TCT TGG ACC |
| Filaggrin | Forward | GCA CTC GTC ATG CAG AGA CTT |
|  | Reverse | GAC CCT CGG TTT CCA CTG T |
| GAPDH | Forward | CCC TCA CTG CTG GGG AGT C |
|  | Reverse | CCT AGG CCC CTC CCC TCT T |

Supplementary Table 2. Compound identifiers for molecular docking

| **Ligand Name** | **Compound identifier (CID)** |
| --- | --- |
| Ambrein | 12305858 |
| Ginsenoside Rb1 | 9898279 |
| Ginsenoside Rb2 | 6917976 |
| Ginsenoside Rb3 | 12912363 |
| Ginsenoside Rc | 12855889 |
| Ginsenoside Rd | 11679800 |
| Ginsenoside Re | 441921 |
| Ginsenoside Rg1 | 441923 |
| Ginsenoside Rg2 | 21599924 |
| Ginsenoside Rg3 | 9918693 |
| Ginsenoside Rh2 | 119307 |
| Ginsenoside Rh3 | 20839223 |
| Ginsenoside CK | 9852086 |
| Ginsenoside F2 | 9918692 |
